# Translating Policy into Practice for Community-Based Management of Rheumatoid Arthritis: Targeting Professional Development Needs among Physiotherapists

**DOI:** 10.1155/2012/240689

**Published:** 2012-11-11

**Authors:** Robyn E. Fary, Helen Slater, Jason Chua, Andrew M. Briggs

**Affiliations:** ^1^Curtin Health Innovation Research Institute, Curtin University, Building 609, 5 Parker Place, Bentley, P.O. Box U1987, Perth, WA 6845, Australia; ^2^School of Physiotherapy, Curtin University, Building 408, Kent Street, Bentley, WA 6102, Australia; ^3^Department of Health, Government of Western Australia, 189 Royal Street, East Perth, WA 6104, Australia

## Abstract

*Introduction*. Contemporary health policy promotes delivery of community-based health services to people with musculoskeletal conditions, including rheumatoid arthritis (RA). This emphasis requires a skilled workforce to deliver safe, effective care. We aimed to explore physiotherapy workforce readiness to co-manage consumers with RA by determining the RA-specific professional development (PD) needs in relation to work and educational characteristics of physiotherapists in Western Australia (WA). *Methods*. An e-survey was sent to physiotherapists regarding their confidence in co-managing people with RA and their PD needs. Data including years of clinical experience, current RA clinical caseload, professional qualifications, and primary clinical area of practice were collected. *Results*. 273 physiotherapists completed the survey. Overall confidence in managing people with RA was low (22.7–58.2%) and need for PD was high (45.1–95.2%). Physiotherapists with greater years of clinical experience, a caseload of consumers with RA, postgraduate qualifications in musculoskeletal physiotherapy, or who worked in the musculoskeletal area were more confident in managing people with RA and less likely to need PD. Online and face-to-face formats were preferred modes of PD delivery. *Discussion*. To enable community-based RA service delivery to be effectively established, subgroups within the current physiotherapy workforce require upskilling in the evidence-based management of consumers with RA.

## 1. Introduction

Rheumatoid arthritis (RA) is an autoimmune disorder associated with systemic inflammation and considerable societal and personal health burdens [1]. The evidence for physiotherapy to address impairment and disability associated with RA is well established [2, 3] and is recognised in clinical guidelines as an important component of overall effective management [4–10]. Recent projections point to a doubling in the prevalence of RA in Australia by 2050 [11]. This highlights the need to implement alternative and sustainable models of health service delivery to consumers with RA in order to maintain service quality and meet projected increasing demand, particularly in light of increasing service pressures being imposed on public tertiary hospital services.

Contemporary health policy for care of consumers with chronic diseases, such as RA, promotes models of care where primary and tertiary care systems articulate to streamline health service delivery [12–15]. These service models emphasise the need to provide clinical services in community-based settings close to where people live, while the need and efficacy of providing community-based physiotherapy to consumers with RA has already been established [2, 3, 16, 17]. In Australia, this means clinical services being potentially provided by physiotherapists working in the private sector, within community-based health facilities, or via virtual communities of practice using technologies such as telehealth to enable access for those consumers living in remote areas. 

To effectively implement contemporary health policy and optimise the delivery of services for consumers with RA and chronic diseases generally, it requires, as a central platform, a skilled health workforce [13, 18, 19]. However, it is well documented that gaps exist in the knowledge and skills required by primary care health professionals to effectively manage consumers with RA and musculoskeletal health generally [20–23]. In the United Kingdom, key areas of rheumatology education for undergraduate health professionals, including physiotherapists, are limited [24]. British and Dutch nurses have similarly reported a need to improve their knowledge and practice of managing RA-related fatigue [25]. In Australia, a recent survey of physiotherapists highlighted a self-reported lack of confidence in recognising the early signs of RA, in knowledge of the disease course, and in evidence-based interventions and the ability to manage a person with RA over the course of the disease [4]. Respondents in the Australian study indicated a clear need for professional development (PD) with over 70% of respondents stating a need in all skills areas surveyed and the same percentage stating a need for PD in 10 of the 12 knowledge areas surveyed. Upskilling the physiotherapy workforce to provide health services in primary care seems an obvious strategy to lever improvements in service delivery in RA and is supported by evidence for improved workforce participation in this clinical area through PD [26].

The learning needs of individual clinicians are likely to vary according to their professional practice and educational history, in particular their experience in musculoskeletal health and practice [27]. Professional development resources may therefore be most effective in enhancing knowledge and clinical skills when delivered in a targeted manner, so that the *right information *is delivered to the* right clinicians*. In order to achieve this, it is important to understand the association between PD needs, and professional and educational characteristics. To ensure that PD resources are delivered in the *right way*, it is also important to understand preferred modes of delivery [28, 29]. The aim of this study was to identify whether physiotherapists' PD needs and confidence in clinical service delivery for consumers with RA were related to total years of clinical experience, current practice experience with consumers with RA, clinical qualifications in musculoskeletal physiotherapy (MSKP), and current primary area of clinical practice in MSKP. A secondary aim was to explore physiotherapists' preferred mode of delivery for PD initiatives. This paper represents a secondary and extended analysis of PD needs data previously collected [4].

## 2. Methods

### 2.1. Survey Development

A web-based survey was developed by one of the authors (JC) using the Qualtrics platform (http://www.qualtrics.com/) to collect, from registered physiotherapists, self-reported information including: (i) demographic, educational and professional characteristics, (ii) confidence in managing consumers with RA across the disease course, and (iii) PD needs in the area of providing clinical physiotherapy services to consumers with RA. 

#### 2.1.1. Demographic, Educational, and Professional Characteristics Data

Survey items included gender, age (years), place of employment (of 6 possible categories), overall years of clinical physiotherapy practice, current clinical caseload involving consumers with RA (yes/no), the percentage of current clinical caseload involving consumers with RA, qualifications higher than foundation physiotherapy degree (of 9 possible qualifications), primary clinical area of practice (of 10 possible areas), and utilisation of government-funded Chronic Disease Management (CDM) plans in their place of employment (yes/no). These management plans are developed by general practitioners and enable consumers with chronic health conditions to access limited government-subsidised allied health services from the private sector. 

#### 2.1.2. Confidence in Managing Consumers with RA across the Disease Course

Self-reported confidence in the areas of (i) early RA detection, (ii) knowledge of the typical clinical course of RA, (iii) knowledge of evidence-based physiotherapy intervention, and (iv) knowledge to effectively and safely manage a consumer with RA throughout the various stages of the disease was assessed by asking four dichotomous (yes/no) questions. 

#### 2.1.3. PD Needs

Survey items regarding physiotherapists' PD needs were developed from results of an earlier study where an international Delphi panel was convened to identify and establish consensus on the essential disease-specific knowledge and clinical skills required by community-based physiotherapists to safely and effectively manage people with RA [4]. That study established consensus among the Delphi panellists on 12 knowledge and 13 skills themes which informed the PD needs items for the survey (these themes are summarised in results of Tables 3 and 4). Responses to the PD needs items were collected using nominal response categories (definitely require PD; may benefit from PD; PD not required). Participants were also asked to indicate their preferred modes of receiving PD (DVD; online; face-to-face; other). Respondents were able to select multiple delivery modes. The survey was tested for technical functionality by its designer (JC) and then pilot tested among a group of five physiotherapists prior to dissemination. The designer used the “test survey” function in Qualtrics to test the functionality of the survey and any skip logic embedded in the survey design. The “test survey” function operates by the software automatically responding to the survey questions using random responses for both nominal response questions and free-text response questions. Designers can select the number of times the software runs the “test survey” function; in this case, the survey was tested *n* = 20 times. The physiotherapists involved in the pilot testing were each provided with a hyperlink to the survey platform, contained within an email. The physiotherapists were asked to undertake the survey and report on its functionality; specifically, the ease in moving between screens and entering and changing responses to survey items. No functional issues were identified during this pilot testing phase and all data entered by the 5 respondents were received as complete survey responses.

### 2.2. Participants

Physiotherapists were eligible to participate in the survey if they were Australian-registered physiotherapists living in Western Australia (WA). 

The e-survey was disseminated in 2011 by the Alumni office of Curtin University (the main educator of physiotherapists in WA) to all physiotherapy graduates from the University for which the Alumni had records (*n* = 680), and by the WA Branch of the Australian Physiotherapy Association (APA) to all its members (*n* = 1550). Both organisations emailed physiotherapy graduates and members of the APA, respectively, using their own databases of contact information. For privacy reasons, the contact details of Alumni and APA members were not provided to the researchers. The email distributed advertised the study, detailed a short blurb regarding the study requirements and inclusion criteria, highlighted the chance of winning a prize for participating, and contained a hyperlink to a unique Qualtrics online survey site where potential participants could enter the survey and respond to further screening questions to determine their eligibility to participate. After a two-week period, a reminder email was sent out again by both organisations to encourage an increased response rate. At no time did the research team have any direct contact with the study sample.

The hyperlink contained in the invitation email sent to potential participants brought them to an online welcome screen, which contained information about the study (title, human research ethics committee approval, target sample, information about a prize, approximate time to complete the survey) and corporate branding from Curtin University and the Department of Health (WA). In total, the online survey consisted of 14–16 screens and 49–57 items (1 introduction screen, 1 consent screen with 1 item, 6–8 screens requiring responses to 14–21 demographic items, and 5 screens requiring responses to 32-33 PD needs items). The number of screens and items varied due to skip logic embedded into the survey design for responses to questions related to demographic characteristics and PD needs. 

Responses to questions requiring a nominal response could be made using mouse clicks in the appropriate area (radio buttons or check boxes), while those requiring a free-text response could be typed into a text field with an uncapped character limit. Users were able to advance to the next screen in the survey by selecting a “next” icon, only when responses to all questions were completed. This mandatory response function ensured that submitted questionnaires had no missing data. Thus, data used in this study were derived only from e-surveys which were submitted as 100% completed by respondents. Users were able to review and modify their answers in preceding screens by selecting the “previous” icon. Responses entered on the screen prior to navigating backwards through the survey were automatically saved. No other response-dependent features or review functions were built into the survey. 

Data concerning date and time of completion of the survey were also captured by the survey. No responses were excluded on the basis of atypical time stamps; however, any responses submitted after the advertised close date of the survey (27 October 2011) were excluded.

In 2011, there were 2600 physiotherapists registered in WA. The risk for duplicate responses stemming from physiotherapists receiving the invitation to complete the survey on more than one occasion was minimised by explicitly asking respondents to complete the survey once only. Further, the Qualtrics platform used browser cookies to block multiple submissions from the same computer. Therefore, while users of computers sharing the same IP address could respond to the survey, an individual user, defined by their computer login details, could not complete the survey more than once without deleting their internet cookies. As an incentive to respond to the e-survey, a prize of one annual membership subscription to the APA was offered.

The study was approved by the Curtin University Human Research Ethics Committee.

### 2.3. Data Analysis

Descriptive statistics were calculated for demographic, professional, and educational characteristics, confidence and PD needs, and preferred mode of PD delivery responses. The PD need dependent variable was collapsed into *PD required *or* PD not required*. Chi-square analysis was used to examine the associations between confidence and PD needs responses and the identified likely binomial professional and educational characteristics correlates to these responses including, current caseload that included consumers with RA, postgraduate qualifications in MSKP, and primary clinical area of MSKP. Logistic regression was used to determine the association between confidence and need for PD responses and total years of clinical experience. Odds ratios and 95% confidence intervals (95% CIs) were calculated to indicate the strength and direction of any association. Analyses were performed using IBM SPSS Statistics, version 19.0.

## 3. Results

Of the 384 e-surveys started online, 285 (81.9%) met the selection criteria and 273 (78.4%) were fully completed and used for data analysis.  Table 1 provides a summary of demographic, professional, and educational characteristic e-survey responses. The most common primary clinical area of practice was in MSKP (49.8%), the most common place of primary employment was in community-based private practice (45.1%) and 50.2% of respondents had a current caseload that included people with RA. Government-funded CDM plans were accepted at 72.5% of the private practices where respondents worked.

Self-reported confidence was low overall, ranging from 22.7% (knowledge of evidence-based physiotherapy interventions) to 58.2% (recognising signs and symptoms of early RA presentation) (Table 2). Multiple significant associations were evident between confidence and the educational and professional characteristic variables (Table 2). Specifically, having greater years of clinical practice experience, a current caseload of consumers with RA, postgraduate MSKP qualifications and, working primarily in MSKP were associated with having confidence in managing consumers with RA. Physiotherapists with postgraduate qualifications in MSKP were 4 times more confident than their colleagues in their knowledge to effectively and safely manage a person with RA throughout the disease stages, 3.68 times more confident in being able to recognise the signs and symptoms of early RA presentation and 3.49 times more confident in their knowledge of the typical clinical course of RA (Table 2). 

The reported need for PD was very high, ranging from 45.1% to 93.4% in knowledge areas and 71.1% to 95.2% in skills areas (Tables 3 and 4). The greatest number of significant associations between PD and the educational and professional characteristic variables was with years of clinical experience. The more years of clinical experience a physiotherapist had, the less likely was their self-reported need for PD, ranging from 5 to 55% less likely to require PD for each 10 years of experience. While there were similar associations between having a current caseload of people with RA, postgraduate qualifications in MSKP and working primarily in MSKP, and the need for PD in both knowledge and skills areas (Tables 3 and 4), they were fewer.

One hundred and eighty respondents (66%) reported that they would be either *interested* or *very interested* in accessing PD related to RA. Of these, 140 (77.8%) selected online delivery, 108 (60.0%) selected face-to-face opportunities, 83 (46.0%) selected DVD, and 4 (2.2%) selected written resources as their preferred modes of PD delivery. There were 106 (58.9%) respondents who selected two or more modes.

## 4. Discussion 

A self-reported lack of confidence and a substantial need for PD in the area of evidence-based management of RA amongst physiotherapists in WA argue for targeted PD in this workforce. In order to upskill physiotherapists to effectively and safely deliver community-based health services for consumers with RA, this PD would ideally be presented using various flexible delivery modes. 

Strong associations were noted between confidence and the variables: years of clinical experience, current RA caseload, postgraduate qualifications in MSKP, and primary work area in MSKP. While these data suggest that physiotherapists with these educational and clinical attributes are less likely to need PD, overall confidence in the management of consumers with RA was very low. Specifically, only 1 in 5 of all respondents reported having confidence in their knowledge of evidence-based physiotherapy interventions and 1 in 3 in their knowledge to effectively and safely manage a consumer with RA throughout the course of the disease. These data highlight a gap in the self-perceived capability of the current physiotherapy workforce in WA necessary to meet the escalating needs of consumers with RA and also reflect similar findings from previous studies. For example, Matheny et al. [30] reported relatively low self-reported confidence amongst family practice residents in the management of musculoskeletal conditions compared to management of nonmusculoskeletal conditions, while Cook et al. [31] noted up to 23.8% of orthopaedic physical therapists reported a lack of confidence in assessing cervical and lumbar spine instability. Furthermore, 50% of respondents in our study lacked confidence in their knowledge of the typical clinical course of RA. As the early identification and intervention of RA is critical to arresting the rapid progression of the disease and optimising responses to therapies [32], addressing this particular issue as a key aspect of any PD must be considered a high priority. Having confidence in these critical knowledge areas is particularly important because community-based physiotherapists are often primary contact clinicians in the Australian healthcare system. 

The least confidence was reported in evidence-based physiotherapy interventions for RA. This finding probably reflects complex and interacting factors such as inadequate emphasis on evidence-based practice within undergraduate Australian physiotherapy curricula in the area of rheumatology, as similarly noted in the UK [24, 33]; outdated or absent awareness of current best-evidence practice; limited exposure to consumers with RA; or possibly that clinical skills in this area are imparted to trainees devoid of a framework incorporating appropriate evidence with clinical reasoning [34]. Despite a range of clinical guidelines [4, 5, 7–9] and Cochrane reviews [35–40] that provide evidence for physiotherapy interventions in RA, uptake of such resources among clinicians is typically low [41, 42], highlighting the challenge of implementing clinical guidelines in the real world [43]. Here, the importance of providing evidence-based, clinically oriented, and flexible learning opportunities for clinicians is emphasized. For example, adopting an intervention that aligns evidence-based practice within a policy and practice framework to upskill primary care practitioners can significantly increase practitioner confidence as documented for the management of low back pain [44]. This policy-to-practice framework could be adapted for the PD needs in RA as identified in the current study.

Confidence in knowledge of evidence-based interventions for RA was no greater for clinicians with postgraduate qualifications in MSKP than those clinicians without this training. While this finding appears paradoxical given the clear association identified between this variable and confidence in treatment effectiveness and safety, one interpretation may relate to the more comprehensive clinical reasoning framework that underpins postgraduate musculoskeletal training in Australia [45]. In this context, it would be anticipated that, regardless of familiarity with clinical guidelines, those with a more robust problem-solving framework would have more confidence in recognising when management was indicated and, more importantly, when it was contraindicated. Furthermore, confidence in knowledge of clinical guidelines is a very explicit and disease-domain specific question; therefore, these divergent responses are understandable. An easily accessible repository of clinically-oriented evidence (i.e., both knowledge and skills-based and one that can be implemented in a real world clinical situation with case studies) may be a very useful tool to bridge this translational evidence-practice gap [46, 47]. 

Physiotherapists with greater number of years of clinical practice experience reported being less likely to need PD across the 12 essential knowledge and 13 essential skills themes. Experienced clinicians possibly have experiential knowledge and skills over a broader patient mix and, like those with postgraduate MSKP qualifications, may have developed a more robust problem-solving framework. Similarly, clinicians with a longer history of clinical practice experience, a caseload consisting of consumers with RA, having postgraduate training in MSKP, or working primarily in MSKP are less likely to require PD in the area of contraindications for physiotherapy, suggesting that the safety aspect of managing consumers with RA is better understood by clinicians with these attributes. To ensure safety of service delivery for early career clinicians without these attributes, PD for this aspect would be a learning area of great importance. 

Only 4 themes had a significant association with all 4 of the educational and professional characteristic variables (2 knowledge and 2 skills themes), confirming that, irrespective of training and work history, there remains a high need for PD in this specific clinical area. Very importantly, there was a greater reported need for *skills* PD than for *knowledge* PD highlighting a potential gap in the translation of acquired knowledge and skills into clinical practice, often coined, the “know-do” gap. In particular, the skills to monitor disease activity and severity are critical as they affect many other aspects of RA management [48]. Flexible modes of PD delivery may be part of a solution enabling the translation of knowledge and skills to enhance clinical practice. In this regard, respondents in our study preferred a variety of PD delivery modes with a strong preference for online access. The high number of respondents choosing online and DVD modes may reflect the reality of geographic isolation in many areas of WA, as well as the reality of time constraints on busy clinicians. However, effectively delivering skills (the “doing”), PD also requires “hands-on” practice and this probably explains the 60% of respondents who also selected face-to-face education as a delivery mode of choice. Moreover, the “know-do” gap may also be more effectively addressed by incorporating clinical practice scenarios into PD, such as clinical vignettes [49]. 

Our findings may also reflect gaps in the rheumatology educational pathway of a physiotherapist. While no data exist within the Australian context, glimpses of such gaps come from UK data [24, 33]. Limited opportunities for Australian undergraduate and postgraduate students to work with people with RA during their training may also compound this training deficiency. There is a similar lack of opportunity for qualified physiotherapists to work in interprofessional teams in primary care, an important feature of effective management for people with RA [4], because most are currently treated within the tertiary hospital sector. However, a willingness to engage physiotherapists in primary care co-management for consumers with chronic diseases is evident given that 72.5% of private practices in our study have access to funded CDM plans. 

Some aspects of our study require further consideration when interpreting the results. A strength of this study is that the majority of the sample currently works within the primary health care sector, suggesting that our results can reasonably be generalised to the broader Australian physiotherapy population. Furthermore, the knowledge and skills items are informed by a robust Delphi process and review of clinical guidelines [4]. The paper also complies with recommendations from Eysenbach regarding the reporting of results from internet-based e-surveys [50]. Despite the low survey response rate, a finding highlighted for online surveys [51], our sample size is comparable to a similar study of Canadian physiotherapists' views on certification, specialisation, extended role practice, and entry-level training in rheumatology [27]. As the results are based on self-reported responses, our findings cannot be directly aligned with clinical practice behaviour. In future studies, the use of vignettes may more appropriately address this issue [49]. Responder bias is possible as it is unknown whether those with an interest in RA self-selected for survey responses. 

## 5. Conclusion

Upskilling the current community-based physiotherapy workforce in Western Australia is necessary to enable effective and sustainable implementation of health policy, that is, delivering health services to consumers with RA closer to their homes. Targeting RA-related professional development opportunities to community-based physiotherapists with specific educational and work-related characteristics in a flexible manner is more likely to be effective.

## Figures and Tables

**Table 1:** Characteristics of respondents.

Characteristic	PD survey respondents (*n* = 273)
Female; *n* (%)	191 (70.0)
Age; mean ± SD, years	36.9 ± 11.8 range (21–69)
Primary place of employment; *n* (%)	
Private practice	123 (45.1)
Community-based health centre	28 (10.3)
Public hospital (tertiary)	42 (15.4)
Public hospital (nontertiary)	28 (10.3)
University	22 (8.1)
Other	30 (11.0)
Secondary place of employment^a^; *n* (%)	
Private practice	20 (27.0)
Community-based health centre	2 (2.7)
Public hospital (tertiary)	9 (12.2)
Public hospital (nontertiary)	3 (4.1)
University	21 (28.4)
Other	19 (25.7)
Primary clinical area of practice; *n* (%)	
Musculoskeletal	136 (49.8)
Nonclinical	28 (10.3)
Paediatrics	18 (6.6)
Sports	18 (6.6)
Other (clinical e.g., burns, rural generalist, oncology)	17 (6.2)
Neurology	17 (6.2)
Cardiopulmonary/medical	16 (5.8)
Women's health	10 (3.7)
Gerontology	8 (2.9)
Chronic disease/rehabilitation	5 (1.2)
Qualifications higher than foundation physiotherapy degree^b^; *n* (%)	100 (36.6)
Graduate certificate	13 (4.8)
Clinical graduate degree	40 (14.7)
Clinical Masters	28 (10.3)
Nonclinical Graduate Degree	9 (3.3)
Masters by coursework	8 (2.9)
Masters by research	12 (4.4)
Ph.D.	10 (3.7)
Fellowship of the Australian College of Physiotherapists	8 (2.9)
Other	4 (1.5)
Years of overall clinical practice experience; mean ± SD	12.6 ± 11.1 range (1–45)
Current caseload includes people with RA; *n* (%)	137 (50.2)
Percentage current caseload managing consumers with RA^c^; mean ± SD	4.5 ± 6.0
Private practices in which Government-funded Chronic Disease Management plans accepted^d^; *n* (%)	100 (72.5)

^a^ *n* = 74; ^b^a number of respondents held multiple higher qualifications; ^c^ *n* = 137; ^d^ *n* = 138.

**Table 2:** Association between having confidence and the variables of years of clinical practice experience, current caseload of consumers with RA, postgraduate (PG) qualifications in musculoskeletal physiotherapy (MSKP), and primary area of work in MSKP (*n* = 273).

Confidence question topics	Having confidence % of respondents	Yrs of clinical experience^a^ Chi-square (df), *P* value OR (95% CI)	Current RA caseload Chi-square (df), *P* value OR (95% CI)	PG MSKP Quals Chi-square (df), *P* value OR (95% CI)	Primary work area MSKP Chi-square (df), *P* value OR (95% CI)
Recognising signs and symptoms of an early presentation of RA	58.2	**28.1** **(1),** <0.01* **1.91** **(1.47** **:** **2.48) **	2.3 (1), 0.12 1.46 (0.90 : 2.36)	**8.6** **(1),** <0.01* **3.68** **(1.47** **:** **0.24)**	0.0 (1), 0.85 1.05 (0.65 : 1.70)
Knowledge of the typical clinical course of RA	46.9	**11.8** **(1),** <0.01* **1.47** **(1.18** **:** **1.84)**	**18.6** **(1),** <0.01* **2.92** **(1.78** **:** **4.78)**	**10.1** **(1),** <0.01* **3.49** **(1.36** **:** **7.82)**	**4.0** **(1),** 0.04* **1.63** **(1.01** **:** **2.63)**
Knowledge of evidence-based physiotherapy interventions for the management of patients with RA	22.7	1.2 (1), 0.28 1.15 (0.90 : 1.47)	**16.1** **(1),** <0.01* **3.42** **(1.84** **:** **6.37)**	0.1 (1), 0.83 0.91 (0.37 : 2.2)	**12.3** **(1),** <0.01* **2.87** **(1.57** **:** **5.26)**
Knowledge to effectively and safely assist in the management of a patient with RA throughout the various stages of their disease	34.4	**11.0** **(1),** <0.01* **1.46** **(1.17** **:** **1.83)**	**18.4** **(1),** <0.01* **3.10** **(1.83** **:** **5.24)**	**14.2** **(1),** <0.01* **4.0** **(1.87** **:** **8.57)**	**13.0** **(1),** <0.01* **2.56** **(1.53** **:** **4.30)**

^a^OR from the equation represents the increase or decrease in odds for a 10-year increase in clinical experience.

Abbreviations: RA: rheumatoid arthritis; Yrs: years; Quals: qualifications; df: degrees of freedom; OR: odds ratio; CI: confidence interval.

**Table 3:** Association between PD needs for knowledge items and the variables of years of clinical practice experience, current caseload of patients with RA, postgraduate (PG) qualifications in musculoskeletal physiotherapy (MSKP), and primary area of work in MSKP (*n* = 273).

Theme	PD required % of respondents	Yrs of clin exp^a^ Chi-square (df), *P* value OR (95%CI)	Current RA caseload Chi-square (df), *P* value OR (95% CI)	PG MSKP Quals Chi-square (df), *P* value OR (95% CI)	Primary work area MSKP Chi-square (df), *P* value OR (95% CI)
Importance of monitoring RA disease activity	93.4	**6.4** **(1),** 0.01* **0.95** **(0.91** **:** **0.99)**	3.7 (1), 0.05 0.36 (0.13 : 1.05)	**4.5** **(1),** 0.04* **0.32** **(0.11** **:** **0.97)**	3.9 (1), 0.05 0.36 (0.12 : 1.04)
Red flags requiring immediate attention and medical review	90.1	**10.6** **(1),** <0.01* **0.58** **(0.41** **:** **0.80)**	3.3 (1), 0.07 0.47 (0.20 : 1.08)	**8.7** **(1),** <0.01* **0.27** **(0.11** **:** **0.68)**	**5.1** **(1),** 0.02* **0.38** **(0.16** **:** **0.91)**
General physiotherapeutic principles of treating RA	86.8	**7.6** **(1),** <0.01* **0.66** **(0.49:0.88)**	**4.5** **(1),** 0.03* **0.46** **(0.22** **:** **0.95)**	**6.5** **(1),** 0.01* **0.34** **(0.14** **:** **0.80)**	**4.6** **(1),** 0.03* **0.45** **(0.21** **:** **0.94)**
Alert to presentation that may suggest RA	86.4	**9.8** **(1),** <0.01* **0.63** **(0.47** **:** **0.84)**	0.7 (1), 0.39 0.74 (0.37 : 1.48)	1.9 (1), 0.17 0.53 (0.21 : 1.33)	0.0 (1), 0.88 1.06 (0.53 : 2.11)
Features requiring referral back to GP or rheumatologist	82.1	**18.9** **(1),** <0.00* **0.55** **(0.42** **:** **0.73)**	1.9 (1), 0.16 0.64 (0.34 : 1.20)	1.01 (1), 0.32 0.64 (0.27 : 1.53)	0.6 (1), 0.45 1.27 (0.68 : 2.37)
Contraindications to some physiotherapy treatments specific to people with RA	81.3	**33.9** ** (1),** <0.01* **0.45** **(0.34** **:** **0.60)**	**5.3** **(1),** 0.02* **0.48** ** (0.26** **:** **0.90)**	**17.7** **(1),** <0.01*0.21 **(0.1** **:** **0.46)**	**4.2** **(1),** 0.04* **0.52** **(0.28** **:** **0.98)**
Understanding the impact of comorbidities on physiotherapy intervention	80.6	**6.6** **(1),** 0.01* **0.71** **(0.55** **:** **0.92)**	1.8 (1), 0.18 0.66 (0.36 : 1.21)	1.5 (1), 0.22 0.60 (0.26 : 1.38)	2.0 (1), 0.16 0.65 (0.35 : 1.19)
Physiotherapeutic principles of treating inflamed joints	79.5	**6.8** **(1),** <0.01* **0.71** **(0.55** **:** **0.92)**	**4.3** **(1),** 0.04* **0.53** **(0.29** **:** **0.97)**	3.8 (1), 0.05 0.46 (0.21 : 1.02)	**9.2** **(1),** <0.01* **0.39** **(0.21** **:** **0.73)**
Comprehensive understanding of RA as a chronic disease	72.2	**6.6** **(1),** 0.01* **0.74** **(0.58** **:** **0.93)**	0.0 (1), 0.97 1.01 (0.60 : 1.72)	0.0 (1), 0.94 1.03 (0.46 : 2.34)	0.1 (1), 0.76 0.92 (0.54 : 1.56)
Multidisciplinary teamwork	71.8	2.8 (1), 0.09 0.82 (0.65 : 1.03)	0.5 (1), 0.48 1.21 (0.71 : 2.05)	0.3 (1), 0.59 1.26 (0.54 : 1.93)	2.1 (1), 0.15 1.47 (0.87 : 2.51)
Importance of early referral to a rheumatologist	68.5	**23.1** **(1),** <0.01* **0.57** **(0.45** **:** **0.72)**	3.2 (1), 0.08 0.63 (0.37 : 1.05)	2.08 (1), 0.15 0.58 (0.28 : 1.22)	1.0 (1), 0.32 1.30 (0.78 : 2.17)
Understanding the importance of good communication	45.1	2.5 (1), 0.11 0.84 (0.67 : 1.04)	0.2 (1), 0.68 0.90 (0.56 : 1.46)	0.1 (1), 0.75 0.89 (0.42 : 1.85)	0.44 (1), 0.51 1.18 (0.73: 1.89)

^a^OR from the equation represents the increase or decrease in odds for a 10-year increase in clinical experience.

Abbreviations: RA: rheumatoid arthritis; PD: professional development; Yrs clin exp: years of clinical experience; Quals: qualifications; df: degrees of freedom; OR: odds ratio; CI: confidence interval.

**Table 4:** Association between PD needs for skills items and the variables of years of clinical practice experience, current caseload of patients with RA, postgraduate (PG) qualifications in musculoskeletal physiotherapy (MSKP), and primary area of work in MSKP (*n* = 273).

Theme	PD required % of respondents	Yrs Clin Exp^a^ Chi-square (df), *P* value OR (95% CI)	Current RA caseload Chi-square (df), *P* value OR (95% CI)	PG MSKP Quals Chi-square (df), *P* value OR (95% CI)	Primary work area MSKP Chi-square (df), *P* value OR (95% CI)
Ability to conduct and record a thorough musculoskeletal examination specific to RA	95.2	**6.5** **(1),** 0.01* **0.55** **(0.35** **:** **0.87)**	3.9 (1), 0.05 0.29 (0.08 : 1.07)	1.6 (1), 0.21 0.44 (0.11 : 1.67)	2.1 (1), 0.15 0.42 (0.13 : 1.41)
Ongoing monitoring of disease activity and severity	93.8	**6.2** **(1),** 0.01* **0.60** **(0.40** **:** **0.89)**	**5.0** **(1),** 0.03* **0.29** **(0.09** **:** **0.91)**	**5.1** **(1),** 0.02* **0.30** **(0.10** **:** **0.90)**	**5.2** **(1),** 0.02* **0.29** **(0.09** **:** **0.90)**
Ability to implement staged treatment strategies in accordance with evidence-based guidelines	91.9	**4.4** **(1),** 0.04* **0.68** **(0.47** **:** **0.97)**	1.7 (1), 0.19 0.55 (0.22 : 1.36)	2.6 (1), 0.11 0.43 (0.15 : 1.25)	0.2 (1), 0.64 0.81 (0.34 : 1.95)
Ability to monitor a patient's progress and outcomes	91.6	**12.8** **(1),** <0.01* **0.52** **(0.37** **:** **0.75)**	3.8 (1), 0.05 0.41 (0.16 : 1.03)	2.2 (1), 0.14 0.45 (0.16 : 1.32)	2.4 (1), 0.12 0.50 (0.21 : 1.22)
Ability to provide education	89.4	0.5 (1), 0.49 0.89 (0.64 : 1.24)	3.1 (1), 0.08 0.49 (0.22 : 1.10)	0.8 (1), 0.37 0.62 (0.22 : 1.76)	1.0 (1), 0.32 0.67 (0.31 : 1.47)
Ability to adjust assessment and treatment strategies in accordance with evidence-based guidelines and comorbidities	89.0	**10.2** **(1),** <0.01* **0.60** **(0.43** **:** **0.82)**	3.7 (1), 0.06 0.46 (0.21 : 1.03)	4.0 (1), 0.05 0.39 (0.15 : 1.00)	1.4 (1), 0.24 0.63 (0.29 : 1.36)
Ability to implement evidence-based treatments while waiting for a diagnosis to be confirmed	86.1	**4.0** **(1),** 0.04* **0.74** **(0.56** **:** **0.99)**	**5.9** **(1),** 0.02* **0.41** **(0.20** **:** **0.86)**	**5.6** **(1),** 0.02* **0.37** **(0.16** **:** **0.87)**	**8.0** **(1),** <0.01* **0.35** **(0.17** **:** **0.74)**
Identification of potential physical complications of RA	85.7	**17.1** **(1),** <0.01* **0.54** **(0.41** **:** **0.73)**	**5.0** **(1),** 0.03* 0.45 **(0.22** **:** **0.92)**	**7.9** **(1),** <0.01* **0.32** **(0.14** **:** **0.73)**	2.5 (1), 0.11 0.57 (0.29 : 1.15)
Ability to take and record a thorough patient history specific to RA	84.6	**5.8** **(1),** 0.02* **0.71** **(0.54** **:** **0.94)**	1.7 (1), 0.19 0.64 (0.33 : 1.25)	**9.3** **(1),** <0.01* **0.30** **(0.13** **:** **0.67)**	1.1 (1), 0.30 0.71 (0.36 : 1.37)
Capacity to be involved with annual multidisciplinary team reviews of patients with RA	81.7	0.60 (1), 0.44 0.90 (0.69 : 1.18)	0.8 (1), 0.36 0.75 (0.41 : 1.40)	2.1 (1), 0.16 0.55 (0.24 : 1.27)	0.4 (1), 0.55 1.21 (0.65 : 2.23)
Excellent communication skills	72.5	3.2 (1), 0.08 0.81 (0.64 : 1.02)	0.83 (1), 0.36 0.78 (0.46 : 1.33)	0.8 (1), 0.39 1.47 (0.61 : 3.54)	0.0 (1), 0.92 1.03 (0.60 : 1.75)
Ability to recognise professional limitations specific to management of RA	71.1	**4.6** **(1),** 0.01* **0.74** **(0.59** **:** **0.93)**	3.9 (1), 0.05 0.59 (0.35 : 1.00)	1.0 (1), 0.32 0.68 (0.32 : 1.45)	0.1 (1), 0.72 1.1 (0.65 : 1.90)
Ability to provide self-management support and encouragement	71.1	2.6 (1), 0.11 0.83 (0.66 : 1.04)	3.9 (1), 0.05 0.59 (0.35 : 1.00)	0.0 (1), 0.85 0.93 (0.42 : 1.05)	1.4 (1), 0.25 1.37 (0.81 : 2.3)

^a^OR from the equation represents the increase or decrease in odds for a 10-year increase in clinical experience.

Abbreviations: RA: rheumatoid arthritis; PD: professional development; Yrs clin exp: years of clinical experience; Quals: qualifications; df: degrees of freedom; OR: odds ratio; CI: confidence interval.
